# Contribution of Inhibitory Metabolites and Competition for Nutrients to Colonization Resistance against *Clostridioides difficile* by Commensal *Clostridium*

**DOI:** 10.3390/microorganisms9020371

**Published:** 2021-02-12

**Authors:** Amber D. Reed, Casey M. Theriot

**Affiliations:** Department of Population Health and Pathobiology, College of Veterinary Medicine, North Carolina State University, Raleigh, NC 27606, USA; adreed2@ncsu.edu

**Keywords:** *Clostridioides difficile*, *Clostridium scindens*, secondary bile acids, deconjugation, dehydroxylation, epimerization, short-chain fatty acids, proline, hydroxyproline

## Abstract

*Clostridioides difficile* is an anaerobic pathogen that causes significant morbidity and mortality. Understanding the mechanisms of colonization resistance against *C. difficile* is important for elucidating the mechanisms by which *C. difficile* is able to colonize the gut after antibiotics. Commensal *Clostridium* play a key role in colonization resistance. They are able to modify bile acids which alter the *C. difficile* life cycle. Commensal *Clostridium* also produce other inhibitory metabolites including antimicrobials and short chain fatty acids. They also compete with *C. difficile* for vital nutrients such as proline. Understanding the mechanistic effects that these metabolites have on *C. difficile* and other gut pathogens is important for the development of new therapeutics against *C. difficile* infection (CDI), which are urgently needed.

## 1. Introduction

*Clostridioides difficile* is an anaerobic, spore-forming, toxigenic bacterial pathogen that was first isolated from the stool of newborn infants in 1935 [1]. *C. difficile* infection (CDI) is the cause of significant morbidity and mortality and is responsible for over 4.8 billion dollars in excess medical costs yearly [2,3]. While the current first line treatment of vancomycin is capable of resolving CDI, 20–30% of patients will experience a recurrence within 30 days. Additionally, 40–60% of patients who experience recurrent CDI once will have multiple recurrences [4,5]. The use of antibiotics, including vancomycin, is a significant risk factor for CDI due to its ability to alter the gut microbiota, resulting in a loss of colonization resistance against *C. difficile* [6,7,8]. Colonization resistance is defined as the ability of the indigenous gut microbiota to protect against colonization by pathogens such as *C. difficile* [9]. Understanding the mechanisms of colonization resistance against *C. difficile* is important for determining the mechanisms by which *C. difficile* is able to colonize the gut after antibiotics and is important for developing new therapeutics and preventatives for CDI. While there are different mechanisms of colonization resistance, there is evidence that commensal gut bacteria from the genus *Clostridium* may play a key role, especially those capable of producing secondary bile acids, which are inhibitory to *C. difficile* [10,11,12,13,14]. In this review, we highlight how commensal *Clostridium* found in the gut are able to alter colonization resistance against *C. difficile,* with a particular emphasis on the production of secondary bile acids and other inhibitory metabolites, as well as competition for nutrients.

## 2. Primary and Secondary Bile Acids Alter the *C. Difficile* Life Cycle 

Bile acids are important signaling molecules that modulate various metabolic functions, play an essential role in fat digestion, and help shape the gut microbiota [15,16]. Primary, or host-derived, bile acids are synthesized in the liver from cholesterol in a multistep enzymatic process via the classical or alternative pathway [17,18]. The classical pathway generates cholate (CA) and chenodeoxycholate (CDCA), whereas the alternative pathway predominately synthesizes CDCA [18]. Primary bile acids, as well as secondary bile acids, that have gone through enterohepatic circulation are conjugated with either taurine or glycine, which makes them impermeable to cell membranes, permitting higher concentrations of bile acids within bile and the gut [19]. These conjugated bile acids are released into the duodenum in response to food ingestion [18,20]. In the small intestine, conjugated primary bile acids are deconjugated by bile salt hydrolases (BSHs) commonly encoded by gut bacteria [21]. After deconjugation, these primary bile acids are further altered by bacteria in the colon in a myriad of ways to create a diverse pool of secondary, or microbiota-derived bile acids. Common secondary bile acids found in the gut include deoxycholate (DCA) and lithocholate (LCA), which are generated by 7α-dehydroxylation from CA and CDCA, respectively [21,22]. DCA and LCA can be epimerized by hydroxysteroid dehydrogenases (HDSHs), generating such bile acids as ursodeoxycholate (UDCA), iso-DCA (iDCA), and iso-LCA (iLCA) [23]. 

**Figure 1:**
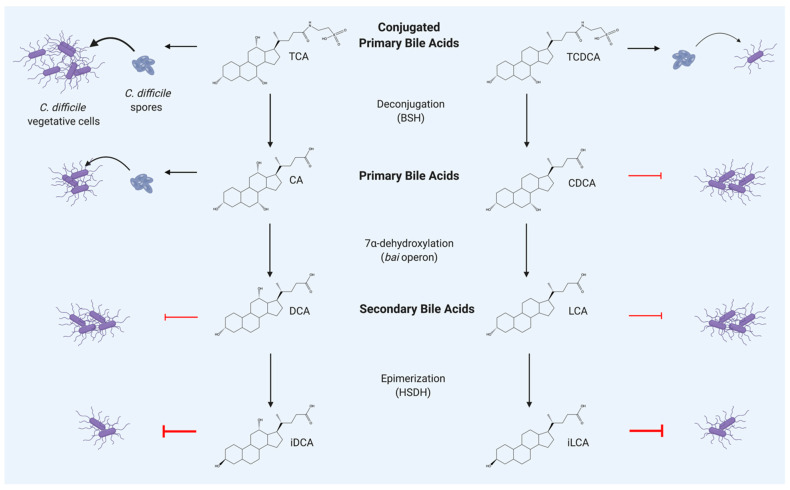
Selected transformations of bile acids carried out by gut bacteria and their effect on *Clostridioides difficile.* TCA is a strong germinant for *C. difficile* spores and TCDCA is a weak germinant. CA is a moderate germinant for *C. difficile* spores, and CDCA inhibits vegetative *C. difficile.* The secondary bile acids DCA and LCA inhibit vegetative *C. difficile* and iDCA and iLCA strongly inhibit *C. difficile.* Abb. BSH, bile salt hydrolase; *bai,* bile acid inducible; HSDH, hydroxysteroid dehydrogenase; TCA, taurocholate; TCDCA, taurochenodeoxycholate; CA, cholate; CDCA, chenodeoxycholate; DCA, deoxycholate; LCA, lithocholate; iDCA, isodeoxycholate; iLCA, isolithocholate. Created with BioRender.com.

Primary and secondary bile acids significantly alter the *C. difficile* life cycle. While bile acids are known to have detergent-like properties that can disrupt bacterial cellular membranes and cause cell lysis, they also affect spore germination, outgrowth, and toxin activity of *C. difficile* in vitro at sub-inhibitory concentrations [24,25,26]. In particular, taurocholate (TCA) is a powerful germinant for *C. difficile* spores, as shown in Figure 1 [25]. Primary bile acids glycocholate (GCA) and CA and the secondary bile acid DCA also stimulate spore germination, while the primary bile acid CDCA, and the secondary bile acids LCA and UCDA inhibit germination of *C. difficile* spores in vitro [24,25,27,28]. Secondary bile acids hyodeoxycholate (HDCA), DCA, iDCA, UDCA, LCA, and iLCA decrease the growth of *C. difficile* in vitro in a dose dependent manner, as shown in Figure 1, and also reduce toxin activity in some strains of *C. difficile* [24,25,29,30]. While the mechanism of how these bile acids alter *C. difficile* has yet to be fully defined, some progress has been made using proteomic approaches. Specifically, when actively growing *C. difficile* is exposed to sub-inhibitory concentrations of CA, DCA, CDCA, or LCA in vitro, the abundance of cell wall binding proteins, cellular chaperones, and cell division proteins increase [26]. When *C. difficile* is grown with sub-inhibitory concentrations of CA, DCA, CDCA, or LCA for a longer period of time, the abundance of alcohol dehydrogenases AdhE1 and AdhE2 decrease, inhibiting the conversion of acetyl-CoA to butynol or ethanol [26]. This indicates that bile acid stress alters the flux through central metabolic pathways of *C. difficile* as well as causing more generalized stress responses. Bile acids also affect enzymes required for Stickland fermentation, which is required for the growth of *C. difficile* and several other bacteria in the genus *Clostridium* [27,28]. Stickland fermentation allows amino acids to be used as an energy source by coupling the oxidation and reduction of paired amino acids to the formation of ATP [29]. Most of the enzymes involved in the reductive Stickland fermentation of leucine to isocaproate increase in abundance when cells are exposed to CA, DCA, CDCA, or LCA [26]. The addition of CA or DCA causes an increased abundance of the proline reductase enzymes PrdA, PrdB, and PrdC, which are required for Stickland fermentation of proline in *C. difficile*, while the addition of CDCA or LCA causes a decreased abundance of those same three enzymes. This indicates that different bile acids can alter *C. difficile* metabolism. Further studies are needed to clarify how specific bile acids are able to shape the formation and activity of proline reductase enzymes, as well as the effect that the altered expression of Stickland fermentation enzymes has on the competitive fitness of *C. difficile.*

Select bile acids can also induce morphological changes in *C. difficile* cells. CDCA, DCA, and LCA cause a significant decrease in the presence of flagella, as well as the flagellar structural protein FliC, and flagellar filaments disappear almost entirely when *C. difficile* is challenged with LCA in vitro [26]. In addition, bacterial cells challenged with CA, DCA, or CDCA were significantly longer than the untreated cells, which is an indicator of bacterial stress, but the addition of LCA does not affect cell shape [26]. DCA causes a significant increase in biofilm formation by *C. difficile* in vitro, whereas LCA does not impact biofilm formation [30]. While CDCA, DCA, and LCA are able to impact toxin activity in *C. difficile* in vitro, the mechanism was unknown until recently [24,31]. Bile acids, including DCA and LCA, bind in a reversible fashion to TcdB, one of the two primary toxins carried by *C. difficile* [31]. LCA and CDCA are able to bind to TcdB with high efficiency and they are able to inhibit cell rounding, a sign of cell death, in human fibroblast cells [31]. DCA binds to TcdB with lower efficiency than LCA and CDCA and does not inhibit cell rounding in human fibroblasts [31]. This binding induced a major conformational change in TcdB, which inhibited the ability of the toxin to bind cell surface receptors of HCT 116 cells, a human colonic cell line [31]. This mechanistic in vitro work demonstrates that bile acids elicit dynamic effects on *C. difficile* and manipulation of the bile acid pool could be a promising therapeutic strategy for treating CDI.

Secondary bile acids are also associated with protection against CDI in mouse models and human subjects [7,10,32,33,34,35,36]. An increase in primary bile acids and a loss of secondary bile acids is observed after treatment with antibiotics and is associated with increased susceptibility to CDI [7,8,32,33,34,35,36]. Cecal extracts from mice made susceptible to CDI stimulate *C. difficile* spore germination, while cecal extracts from mice resistant to CDI inhibit spore germination, indicating that antibiotic-induced changes in bile acid levels in vivo are sufficient to induce germination and outgrowth of *C. difficile* spores [36,37]. However, *C. difficile* spores are able germinate in the small intestine prior to antibiotics, indicating that the bile acids present in the small intestine do not protect against CDI [37]. After human fecal microbiota transplantation (FMT), an increase in microbial diversity is observed and secondary bile acid metabolism is restored [38,39]. Specifically, the levels of secondary bile acids including DCA, LCA, and UCDA are increased and the primary bile acids CA and CDCA are decreased in CDI patients after receiving an FMT [39]. In addition, fecal samples of patients with CDI have a lower prevalence of *baiCD,* a gene present in commensal *Clostridium* required for the synthesis of DCA and LCA via 7α-dehydroxylation, although *baiCD* has also been found in the stool samples of individuals with failed FMTs [12,40]. *Clostridium scindens* is a commensal bacterium found in the gut microbiota [41]. It produces DCA and LCA and is associated with the return of colonization resistance against *C. difficile* in a mouse model of CDI, however *C. scindens* has also been found to be present in the stool samples of individuals with CDI [10,42]. Mice that receive *C. scindens* before being challenged with *C. difficile* show increased levels of LCA, although levels of most other bile acids are unchanged [10]. While manipulation of the bile acid pool using commensal bacteria is a promising strategy, the addition of exogenous bile acids can also affect the progress of CDI. Challenging mice exogenously with the secondary bile acid UDCA attenuates disease early during CDI, and also alters the fecal bile acid metabolome without significantly altering the gut microbiome [43]. 

## 3. Bile Acid Altering Enzymes Encoded by Commensal *Clostridium*

### 3.1. Bile Salt Hydrolases

Bile salt hydrolases (BSHs) are microbial enzymes that deconjugate primary and secondary bile acid from the amino acids they are conjugated to, usually taurine and glycine [44]. While BSHs are commonly encoded by multiple members of the gut microbiota, commensals in the genus *Clostridium* rarely encode BSHs, although *Clostridium hiranonis* and the pathogen *Clostridium perfringens* both encode BSHs and have demonstrated BSH activity [21,45]. *C. hiranonis* is the only bacterium to date known to have the capability for both 7α-dehydroxylation and deconjugation [45]. The presence of BSHs in the gut are hypothesized to be important for several reasons. BSHs are considered the gateway step for the transformation of primary bile acids to secondary bile acids, as further transformations cannot occur until the conjugated amino acid is removed [21]. The taurine or glycine that is released when deconjugation occurs may be acquired for nutrition by members of the gut microbiota [46]. A recent study showed that bile acids can also be conjugated with tyrosine, phenylalanine, or leucine in mice, however deconjugation of these conjugated bile acids by BSHs is unknown at this time [47]. Interestingly, one strain of an unnamed *Clostridium* bacterium capable of deconjugation shows increased growth when taurine was added to the growth medium, indicating that BSH activity might be nutritionally beneficial [48]. Taurine is also enriched in the feces of pediatric inflammatory bowel disease patients with CDI, indicating a potential association between *C. difficile* and taurine [49]. 

In addition, a *bsh* encoded by *Bifidobacterium longum* is transcriptionally coupled to *glnE* (glutamine synthetase adenylyltransferase), which indicates that deconjugation activity may be coupled to nitrogen regulation [50]. However, lactobacilli grown with conjugated bile acids do not utilize the steroid moiety of the bile acid for cellular precursors and taurine does not affect growth, indicating that not all bacteria encoding a *bsh* obtain a direct nutritional benefit from deconjugation [51]. BSH activity has been hypothesized to detoxify conjugated bile acids by converting them to a less toxic form, as the *bsh* encoded by *Listeria monocytogenes* is important for resistance to bile in vitro and is an important virulence factor in animal models [52]. However, unconjugated bile acids are more toxic to some *Lactobacillus* spp. than their conjugated forms, meaning that deconjugation of bile acids can cause an increase in toxicity for at least some members of the gut microbiota [53,54]. Conjugated bile acids are more soluble than deconjugated bile acids, so the increased toxicity observed may be offset by the decreased bioavailability that occurs when micelles form [55,56]. In addition, deconjugation is important for producing free bile acids available for 7α-dehydroxylation [41,57]. BSH activity is also correlated with resistance to *C. difficile* after FMT [58]. Pre-FMT stool samples harbor reduced BSH activity and a lower proportion of BSH-producing bacterial species when compared with donor stool and post-FMT stool. Additionally, mice inoculated with *Escherichia coli* expressing a highly active BSH have a ~70% reduction in *C. difficile* viable counts when compared to mice inoculated with non-BSH expressing *E. coli* [58]. This indicates that BSH activity could be a significant contributor to the efficacy of FMT in treating recurrent CDI. 

### 3.2. Bile Acid Inducible Operon

**Figure 2:**
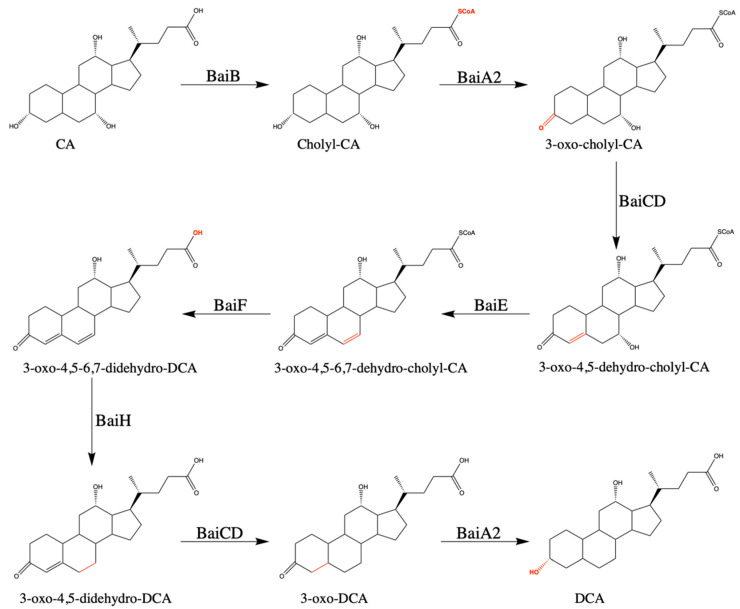
Proposed pathway for conversion from CA to DCA via 7α-dehydroxylation. This metabolic pathway converts CA to DCA or converts CDCA to LCA in eight steps. Abb. BaiB, bile acid-coenzyme A ligase; BaiA2, 3α-hydroxysteroid dehydrogenase; BaiCD, 7α-hydroxy-3-oxo-Δ^4^-cholenoic acid oxidoreductase; BaiE, bile acid 7α-dehydratase; BaiF, bile acid coenzyme A transferase/hydrolase; BaiH, 7β -hydroxy-3-oxo-Δ^4^-cholenoic acid oxidoreductase; CA, cholate; DCA, deoxycholate.

Commensal *Clostridium* that harbor the bile acid inducible (*bai*) operon are capable of synthesizing DCA from CA and LCA from CDCA via 7α-dehydroxylation. While the enzymes responsible for the steps in the oxidative arm of the metabolic pathway have been known for some time, the reductive arm has only recently been defined by reconstructing the pathway in vitro [59]. The proton-dependent transporter BaiG is responsible for transporting the primary unconjugated bile acid into the cell [60]. Six core enzymes encoded by the *bai* operon are sufficient for completing the 7α-dehydroxylation pathway, as shown in Figure 2 [59]. Coenzyme A is ligated onto the substrate in an ATP dependent manner by BaiB and the dehydrogenase BaiA2 oxidizes the 3-hydroxy group [61,62]. The NADH/flavin-dependent oxidoreductase BaiCD catalyzes the formation of the C_4_=C_5_ bond and the 7α-dehydratase BaiE catalyzes the formation of the C_6_=C_7_ bond by removing the 7α hydroxyl group [12,63]. The 7α-dehydration is the last step in the oxidative arm of the pathway and is irreversible and rate limiting [64]. The reductive arm of the 7α-dehydroxylation consists of four steps. The removal of Coenzyme A is catalyzed by the bile acid-CoA hydrolase BaiF, which can also ligate CoA onto the primary unconjugated bile acid in an ATP independent manner [65,66]. The NADH/flavin-dependent oxidoreductases BaiH and BaiCD catalyze the removal of the C_6_=C_7_ and C_4_=C_5_ bonds and BaiA2 performs the final reductive step, catalyzing the transformation from 3-oxo-DCA to DCA [59]. The enzyme responsible for transport of DCA out of the cell has yet to be determined. 

While the enzymes discussed above can sufficiently execute 7α-dehydroxylation, other enzymes are also capable of performing steps in this pathway, indicating some redundancy. Of particular interest is BaiA. While BaiA2 was the enzyme used to reconstruct the 7α-dehydroxylation pathway in vitro, another 3α-hydroxysteroid dehydrogenase called BaiA1 is also present in some of the bacteria that have demonstrated 7α-dehydroxylation capability [61]. BaiA1 is a close homolog of BaiA2 with 92% sequence identity that can also perform the oxidative step in the pathway [61]. BaiA1 has not been shown to catalyze the transformation from 3-oxo-DCA to DCA, but since *Clostridium hylemonae* TN 271 carries *baiA1,* but lacks *baiA2* and has been shown to produce DCA, BaiA1 is likely able to perform both steps in the pathway, as *C. hylemonae* would be unable to produce DCA if *baiA2* was necessary for 7α-dehydroxylation [67]. *Clostridium hiranonis* TO 931 carries *baiA2,* but lacks *baiA1,* while *C. scindens* ATCC 35704 and *C. scindens* VPI 12708 carry both [21]. *C. scindens* VPI 12708 also has a second copy of *baiA1,* referred to as *baiA3* [68]. Another enzyme that is capable of performing steps in the 7α-dehydroxylation pathway is the flavoprotein BaiN, which is capable of converting 3-oxo-4,5-6,7-didehydro-DCA to 3-oxo-4,5-dehydro-DCA and then to 3-oxo-DCA, which are steps that can also be performed by BaiH and BaiCD, respectively [59,69]. 

While all organisms known to carry the *bai* operon have 7α-dehydroxylation activity, the regulation of the *bai* operon has yet to be fully elucidated [45,67,70,71]. *C. scindens* and *C. hylemonae* have increased expression of genes in the *bai* operon in defined media supplemented with CA, and *C. hiranonis* in rich media supplemented with CA [70,71]. While *C. scindens* also has increased expression of selected *bai* operon genes when grown in rich media, *C. hylemonae* does not, indicating differences in regulation of the *bai* operon between commensal *Clostridium* [57]. 

While most of the enzymes involved in 7α-dehydroxylation are not extensively characterized, BaiA and BaiE have both undergone structural and functional characterization [61,72]. The short chain dehydrogenase/reductase BaiA2 as well as the homolog BaiA1 shows exclusive preference for the cofactor NAD(H) rather than NADP(H), likely due to steric hindrance involving Glu42 in the cofactor binding site [61]. The dehydratase BaiE shows a preference for 3-oxo-Δ^4^-CDC-CoA over 3-oxo-Δ^4^-CDCA, with the K_cat_/K_M_ being an order of magnitude higher for the former than the latter, indicating that the 7α-dehydration step is more efficient when the intermediate is ligated to CoA [72]. 

### 3.3. Hydroxysteroid Dehydrogenases

Bacterial hydroxysteroid dehydrogenases (HSDHs) epimerize bile acid hydroxy groups on the 3-, 7-, or 12- carbons of bile acids in a two-step process requiring an α- and a β-HSDH that generates a stable oxo intermediate [21]. Commensal *Clostridium* can encode multiple HSDHs. Commensal *Clostridium* that encode the *bai* operon carry both a 7α-HSDH (*baiA*) as well as a 7β-HSDH [21]. Organisms with a 7α- and a 7β-HSDH can produce UDCA, which is the 7*β*-epimer of CDCA [21]. As UDCA is more hydrophilic and thus less toxic to gut bacteria than CDCA, the epimerization of CDCA using a 7β-HSDH could serve as a survival advantage for bacteria capable of accomplishing this transformation [21,73]. In addition, *Ruminococcus gnavus* carries a 3α-HSDH and *Clostridium innocuum* carries a 3β-HSDH [21,44,74]. The 3α/β epimerization of DCA and LCA creates iDCA and iLCA, respectively, which are the second most abundant secondary bile acids after DCA and LCA [22]. While no bacteria in the genus *Clostridium* have made iDCA, *R. gnavus* uses a 3α-HSDH to create the intermediate of 3-oxoDCA and then a 3β-HSDH to complete the transformation from DCA to iDCA [74]. iDCA exhibits reduced toxicity in vitro to some gut commensals including multiple species of *Bacteroides* and *Clostridium sporogenes* but has the ability to inhibit multiple strains of *C. difficile* at very low concentrations [24,74]. This indicates that the conversion from DCA to iDCA can serve to reduce toxicity for some commensals, as well as assisting the gut microbiota with colonization resistance against pathogens such as *C. difficile.* These same 3α- and 3β-HSDHs convert LCA to iLCA, which inhibits the growth of multiple strains of *C. difficile* in vitro at a lower concentration than LCA [24,74]. The toxicity of iLCA when compared to LCA on various commensals has yet to be determined, but it is possible that the epimerization of LCA to iLCA serves to reduce toxicity for some members of the gut microbiota, as well as assisting with colonization resistance against enteric pathogens such as *C. difficile.*

## 4. Bile Acids, Other Intestinal Pathogens, and the Host 

While bile acids modified by commensal *Clostridium* affect the life cycle of *C. difficile,* they also have an inhibitory effect on other intestinal pathogens as well as a strong effect on the host. Bile acids can induce the transcription of genes responsible for DNA repair and recombination in *E. coli*, *Salmonella enterica* serovar Typhimurium, *Bacillus cereus*, and *L. monocytogenes* [75,76]. Genes responsible for maintaining the integrity of the cellular envelope are also upregulated in *B. cereus* and *L. monocytogenes*, indicating that bile acids damage the bacterial membrane and cellular DNA [76]. In particular, multiple strains of *Shigella* show a significant increase in biofilm formation and 143 genes have differential transcription when exposed to bile salts, which indicates a strong stress response [77]. Enteric pathogens have multiple bile resistance mechanisms including efflux pumps and DNA repair mechanisms, but bile acids are still important in colonization resistance against these intestinal pathogens [76].

Bile acids are important signaling molecules within the host as well. They interact primarily with the G-Protein-Coupled Bile Acid Receptor-1 (GPBAR-1, aka TGR5) and Farnesoid-X-Receptor alpha (FXRα) which belong to the nuclear receptor superfamily [17,18,21]. Secondary bile acids produced by commensal *Clostridium* are potent agonists for TGR5, specifically DCA and LCA [17,78]. TGR5 has been implicated in the regulation of multiple metabolic functions including glucose metabolism and the conversion of fat into energy, making it a potential target for treating obesity [17,78]. The most potent agonist for FXRα is CDCA, but DCA and LCA are also agonists for this receptor [17]. FXRα controls the enterohepatic circulation of bile acids and acts as an anti-inflammatory mediator in the liver and intestine, which could allow it to potentially help prevent tumor development [17]. However, high levels of the secondary bile acids DCA and LCA have been shown to correlate with tumors in the liver and intestine, specifically colon cancer [17,21]. High levels of DCA are also correlated with cholesterol gallstone disease in some patients [21]. The levels of bile acids can also affect FXR receptor expression, as giving mice exogenous UCDA increases the expression of TGR5 and FXR, causing alterations to the bile acid metabolome [43]. 

Bile acids are important not just for their effect on the gut microbiota and their contribution to colonization resistance against *C. difficile* and other intestinal pathogens, they are also important determinants of several other aspects of human health. Further studies examining the rational manipulation of bile acid pools and the effect of this alteration on colonization resistance against *C. difficile* and other intestinal pathogens are necessary and understanding the production of secondary bile acids by commensal *Clostridium* and other microbes is important for advancing our knowledge of human health and disease. 

## 5. Production of Inhibitory Metabolites

While bile acids play a significant role in modulating the composition of the gut microbiota, there are other bacterial metabolites that can affect the gut microbiota and colonization resistance against *C. difficile* such as short-chain fatty acids (SCFAs) [38]. SCFAs are metabolized from fiber by commensal bacteria and the concentration of SCFAs are low in patient stool after taking broad spectrum antibiotics, in CDI patients, and in CDI-susceptible mice [38,79]. Increased levels of SCFAs are correlated with decreased tissue damage and immunomodulatory effects, making rational manipulation of SCFA production a potential strategy for targeted therapeutics against CDI. Increased levels of the SCFAs propionate, succinate, and butyrate were observed after FMT for recurrent CDI [38]. In addition, valerate inhibits *C. difficile* in a chemostat model, and butyrate can protect against *C. difficile*-induced colitis in the murine gut via reducing intestinal permeability and microbial translocation in an HIF-1 dependent fashion [42,80,81,82]. Members of the *Clostridium* cluster XIVa and IV are a significant source of butyrate production in the gut and are significantly depleted in the feces of patients with CDI or with nosocomial diarrhea (*C. difficile* negative) when compared to healthy control samples [80,81,82]. 

Despite the ability of multiple strains of *C. difficile* to generate butyrate, the presence of butyrate in the gut is associated with decreased fitness for *C. difficile* [83]. Specifically, when mice are fed microbiota-accessible carbohydrates, the SCFAs propionate, acetate, and butyrate increase, and the *C. difficile* burden decreases [83]. In addition, all three SCFAs negatively affect the growth of *C. difficile,* although all three SCFAs cause toxin expression to increase in vitro [83]. However, the overall level of toxin decreases due to the lower *C. difficile* burden in mice that are fed diets rich in microbiota-accessible carbohydrates [83].

Butyrate can be produced by bacteria through multiple pathways. The most common pathway in *Clostridium* is the synthesis of butyryl-CoA from acetyl-CoA and the subsequent liberation of butyrate from the CoA molecule [84]. There are multiple arrangements of the butyrate synthesis genes in *Clostridium,* with two arrangements being present in Cluster XIVa and a third distinct arrangement being present in butyrate producing *Clostridium* in Cluster I and Cluster XVI [84]. After butyryl-CoA is produced, the CoA moiety can be removed by butyryl-CoA/acetate CoA transferase (But) or the butyryl-CoA can be phosphorylated by phosphate butyryltransferase (Ptb), then transformed to butyrate by butyrate kinase (Buk), which generates ATP [85]. Most butyrate-producing *Clostridium,* including *C. difficile* contain Buk, some contain But instead, and a small number of strains encode both proteins [85].

However, lysine, glutarate, 4-aminobutyrate, and succinate can also serve as substrates for the production of butyrate. These three pathways are separate from the acetyl-CoA pathway, but all four pathways merge at the energy generating step where crotonyl-CoA is transformed into butyryl-CoA by the Bcd complex [85]. Multiple strains of *C. difficile* can generate butyrate using acetyl-CoA, 4-aminobutyrate, or succinate as a substrate. *Clostridium sticklandii* can use acetyl-CoA or lysine as a substrate [85,86]. The generation of butyrate from succinate by *C. difficile* is of particular interest as the ability to ferment succinate gives *C. difficile* a competitive advantage [86]. 

Antimicrobial compounds produced by members of the gut microbiota also affect colonization resistance against *C. difficile. C. scindens* ATCC 35704 produces 1-acetyl-β-carboline, a tryptophan-derived antibacterial compound that inhibits multiple Gram-positive pathogens found in the gut, including *C. difficile, Staphylococcus aureus*, and *Clostridium sordellii* [87]. While the specific mechanism of action is not known, cell division of *C. difficile* was inhibited and the additional presence of DCA or LCA enhanced the inhibitory effect of 1-acetyl-β-carboline in vitro [87].

## 6. Competition for Nutrients 

Competition for nutrients also plays an important role in colonization resistance against *C. difficile* and other pathogens. Colonization of a susceptible murine host by a nontoxigenic strain of *C. difficile* protects against colonization by toxigenic *C. difficile,* indicating that colonization by bacteria with similar nutritional requirements can protect the host [88,89]. Strains of *C. difficile* belonging to the epidemic ribotypes (RT) 027 and 078 have gained the ability to metabolize low concentrations of trehalose, a common food additive [90]. In the RT 027 strain, a point mutation occurred that increased sensitivity to trehalose, while the RT 078 strain acquired additional genes that metabolize trehalose [90]. While the exact contribution to competitive fitness is unknown, the ability to metabolize trehalose increased virulence in a mouse model of *C. difficile,* indicating that increased ability to compete for trehalose in the gut may provide some form of competitive advantage [90]. *C. difficile* also uses sugar alcohols such as mannitol, N-acetylated amino acids, and carbohydrates during early infection in the murine gut, but the effect of each of those nutrients on competitive fitness is unknown [91].

Proline, hydroxyproline, and glycine are the most efficient electron acceptors for Stickland fermentation, while leucine, isoleucine, and alanine are the most efficient electron donors [27]. *C. difficile* is auxotrophic for isoleucine, leucine, and proline, and proline concentration affects the in vitro expression of genes in the *prd* operon which is responsible for proline reduction in Stickland fermentation [27,92]. Availability of these amino acids (alanine, glycine, leucine, isoleucine, and proline) in the gut correlates with increased susceptibility to CDI in a mouse model [93]. Proline in particular is important for *C. difficile* colonization as a *prdB* mutant is unable to use proline as an energy source. When a *C. difficile prdB* mutant was tested in a mouse model of CDI, the mice challenged with the *prdB* mutant had reduced colonization and a lower concentration of TcdB in their stool when compared to mice challenged with wild type *C difficile,* indicating that the ability to ferment proline is important for colonization and virulence [93]. In addition, when wild type *C. difficile* capable of fermenting proline and a *prdB* mutant were grown in the presence or absence of a commensal clostridia panel, the wild type *C. difficile* had a fitness advantage when the commensals were present, indicating that the presence of commensal clostridia increases reliance on proline fermentation [94]. However, when *C. difficile* competed with *Paeniclostridium* spp. or *Clostridium xylanolyticum,* two members of the commensal clostridia panel able to ferment proline, the competitive advantage conferred by wild type *C. difficile* in comparison to the *prdB* mutant was lower than when it was only competing with bacteria unable to ferment proline [94]. This indicates that *C. difficile* competes with commensal clostridia for proline. *C. difficile* also has a competitive advantage over *C. scindens, C. hylemonae,* and *C. hiranonis* in a rich media, although the extent to which this is due to the ability of *C. difficile* to ferment proline is unknown [57]. 

Hydroxyproline (Hyp) is a derivative of proline which has been post translationally modified by prolyl-4-hydroxylase [95]. It is important for stabilizing the triple helix structure in collagen, the most abundant mammalian protein [96]. It can be converted to proline in a two-step process that requires the hydroxyproline dehydratase HypD as well as the pyrroline-5-carboxylate reductase ProC, both of which are present in *C. difficile* [97,98]. Homologs of HypD are widespread in the gut microbiome, which suggests that the ability of bacteria to reduce hydroxyproline is useful in the gut [97]. However, of the bacteria encoding *hypD,* only a subset had an adjacent *proC* gene, indicating that the ability to reduce hydroxyproline to proline is not ubiquitous [97]. While Stickland fermentation of proline is important for *C. difficile* metabolism, it is not yet known how the reduction of hydroxyproline affects competitive fitness. However, the widespread presence of HypD and the competitive fitness advantage gained by proline fermentation in the presence of commensal clostridia indicates that it may play a significant role in the colonization of *C. difficile* in the gut [94,97]. 

## 7. Conclusions

There are several mechanisms of how the gut microbiota provides colonization resistance against *C. difficile* presented in this review, including the production of inhibitory metabolites, such as secondary bile acids, SCFAs, and antimicrobials, as well as competition for nutrients, especially proline and other amino acids necessary for Stickland fermentation. Understanding the mechanistic effects that these metabolites have on *C. difficile* and other gut pathogens is important for the development of new therapeutics against CDI, which are urgently needed.
